# Supplementary figures and images for: miRNA-27a is essential for bone remodeling by modulating p62-mediated osteoclast signaling
Source: eLife. 2023 Feb 8;12:e79768. doi: 10.7554/eLife.79768 (PMC9946445; doi:10.7554/eLife.79768)

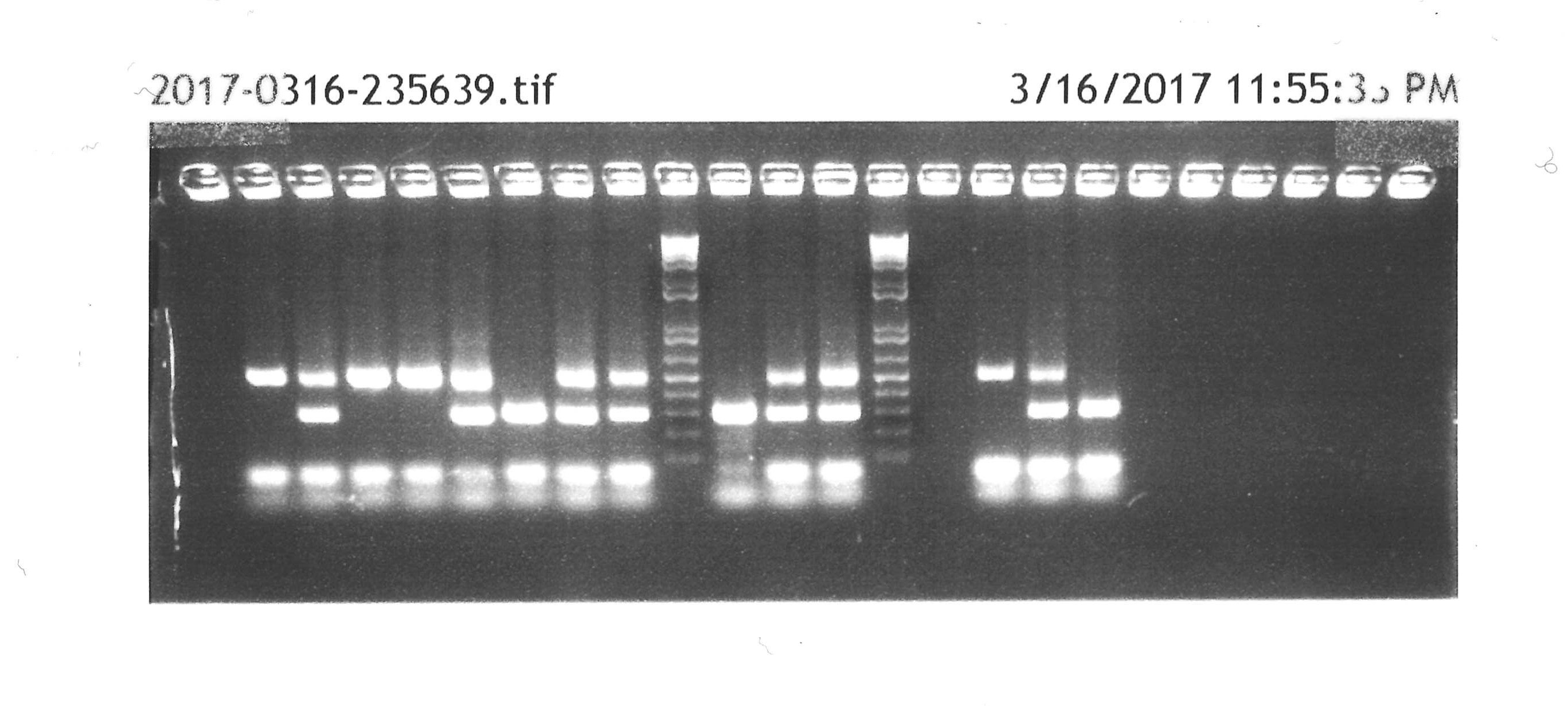

Supplement: Figure 1—source data 2. [file elife-79768-fig1-data2.zip › Figure 1-souce data 2.tif]

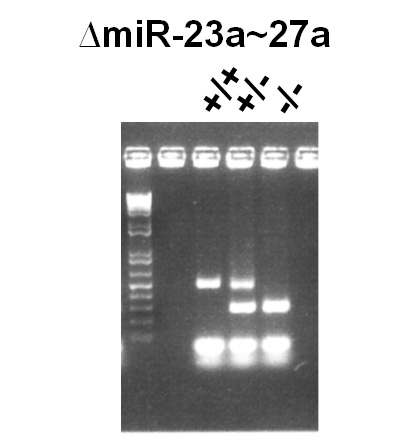

Supplement: Figure 1—source data 3. [file elife-79768-fig1-data3.zip › Figure 1-souce data 3.tif]

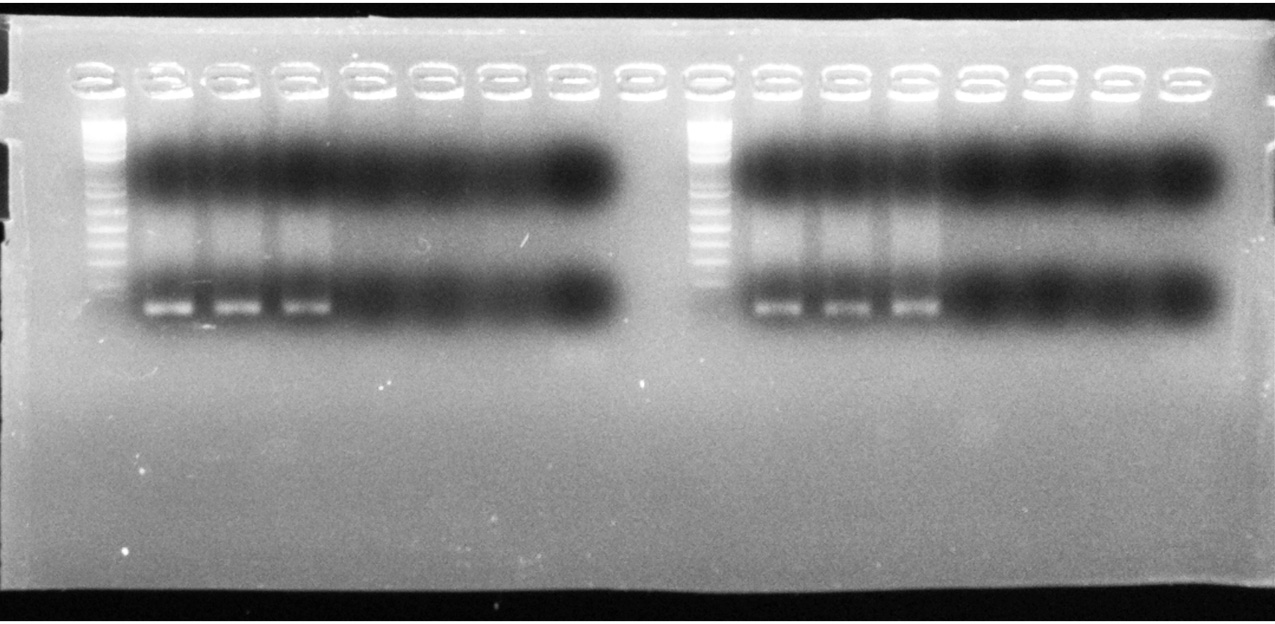

Supplement: Figure 1—source data 4. [file elife-79768-fig1-data4.zip › Figure 1-souce data 4.tif]

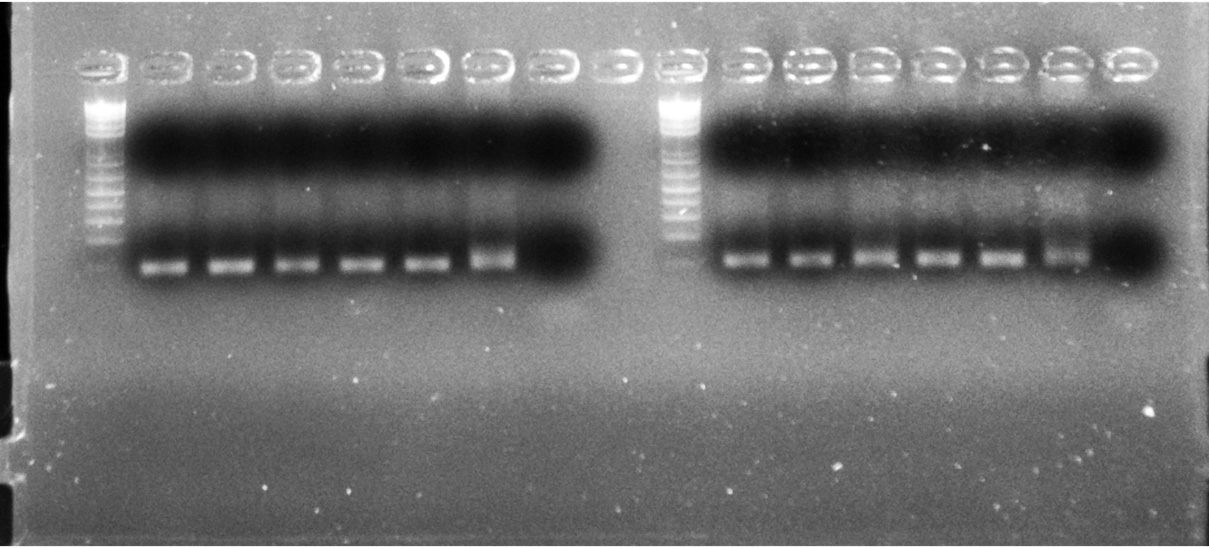

Supplement: Figure 1—source data 5. [file elife-79768-fig1-data5.zip › Figure 1-souce data 5.tif]

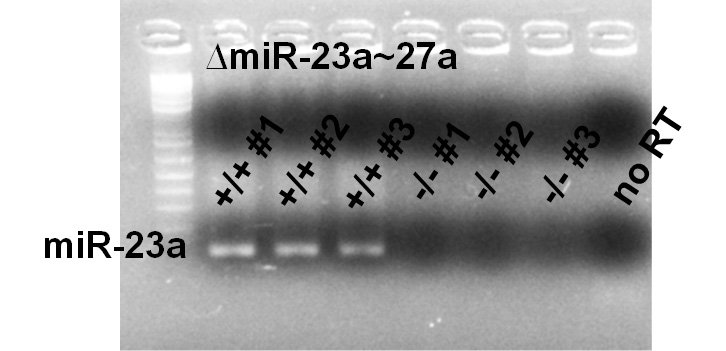

Supplement: Figure 1—source data 6. [file elife-79768-fig1-data6.zip › Figure 1-souce data 6.tif]

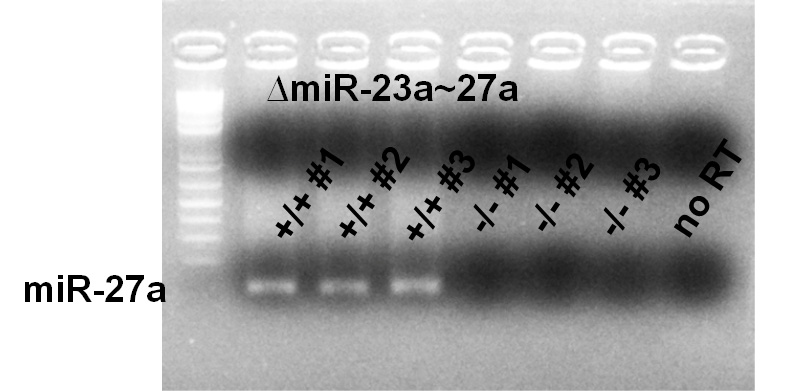

Supplement: Figure 1—source data 7. [file elife-79768-fig1-data7.zip › Figure 1-souce data 7.tif]

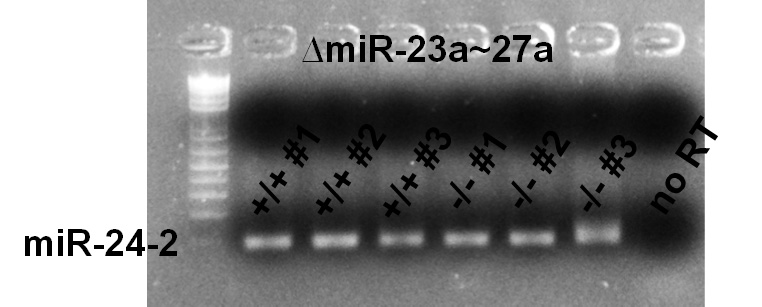

Supplement: Figure 1—source data 8. [file elife-79768-fig1-data8.zip › Figure 1-souce data 8.tif]

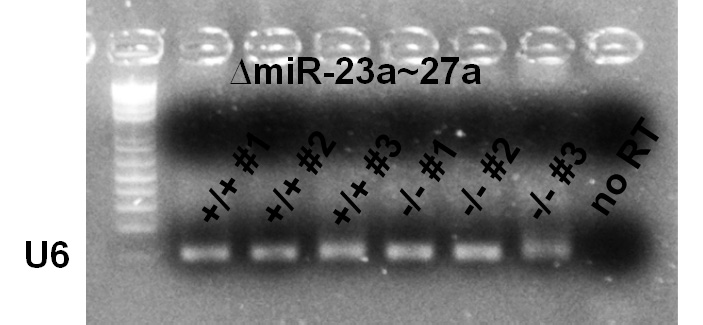

Supplement: Figure 1—source data 9. [file elife-79768-fig1-data9.zip › Figure 1-souce data 9.tif]

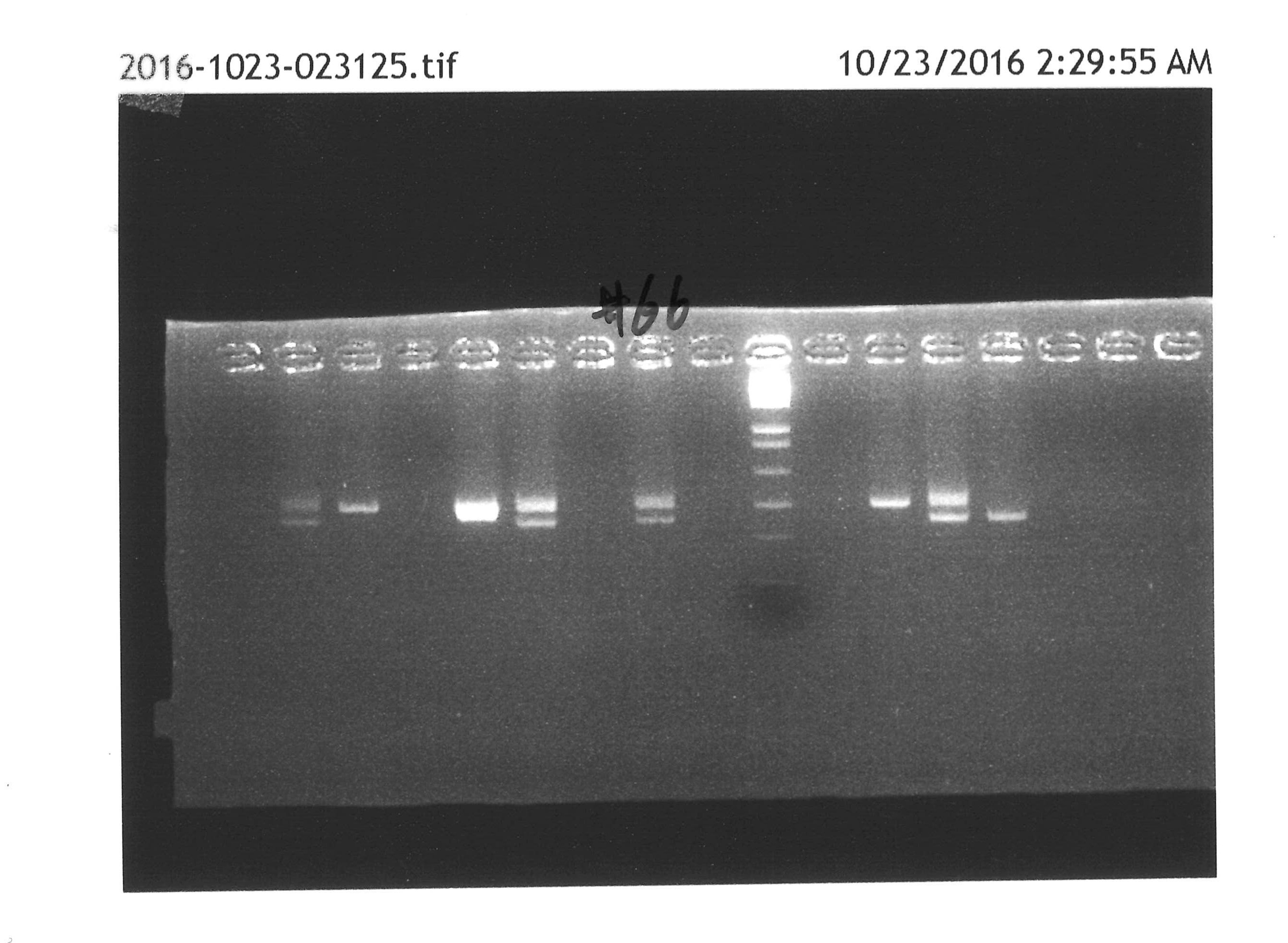

Supplement: Figure 4—source data 2. [file elife-79768-fig4-data2.zip › Figure 4-souce data 2.tif]

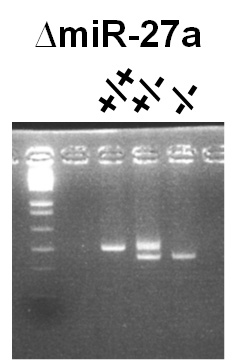

Supplement: Figure 4—source data 3. [file elife-79768-fig4-data3.zip › Figure 4-souce data 3.tif]

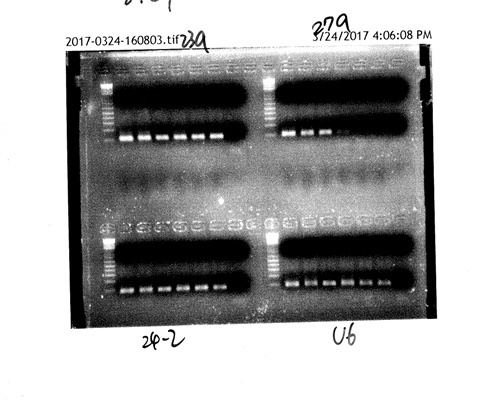

Supplement: Figure 4—source data 4. [file elife-79768-fig4-data4.zip › Figure 4-souce data 4.tif]

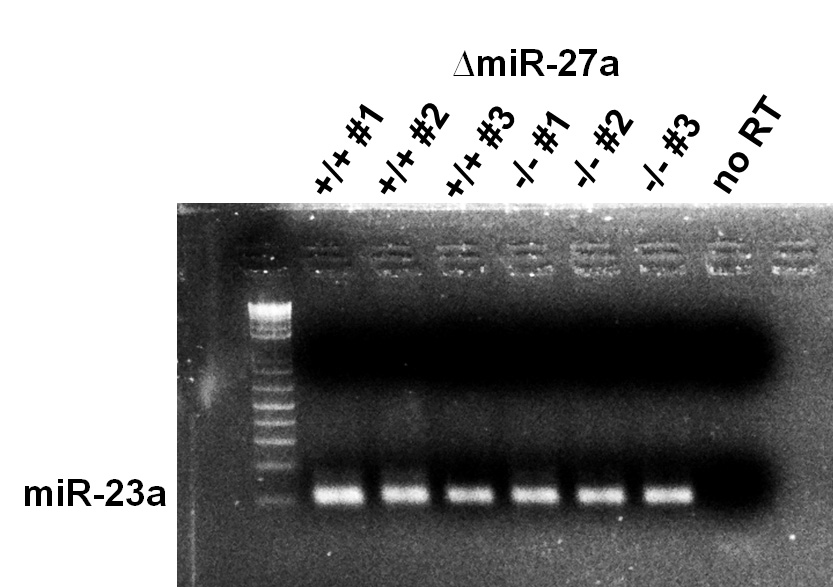

Supplement: Figure 4—source data 5. [file elife-79768-fig4-data5.zip › Figure 4-souce data 5.tif]

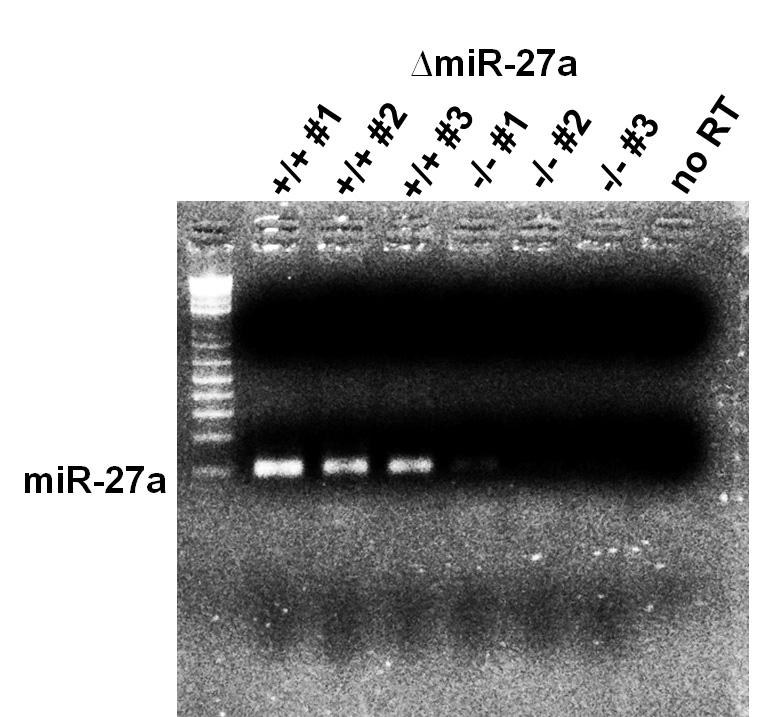

Supplement: Figure 4—source data 6. [file elife-79768-fig4-data6.zip › Figure 4-souce data 6.tif]

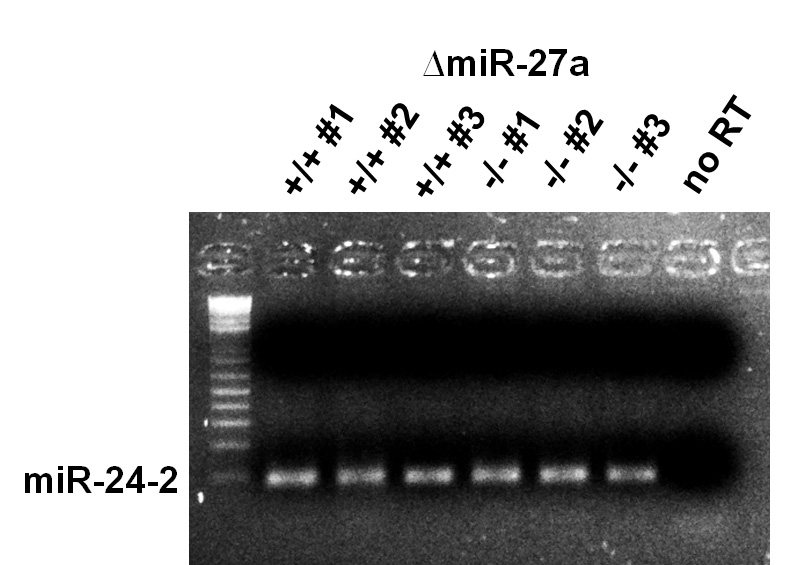

Supplement: Figure 4—source data 7. [file elife-79768-fig4-data7.zip › Figure 4-souce data 7.tif]

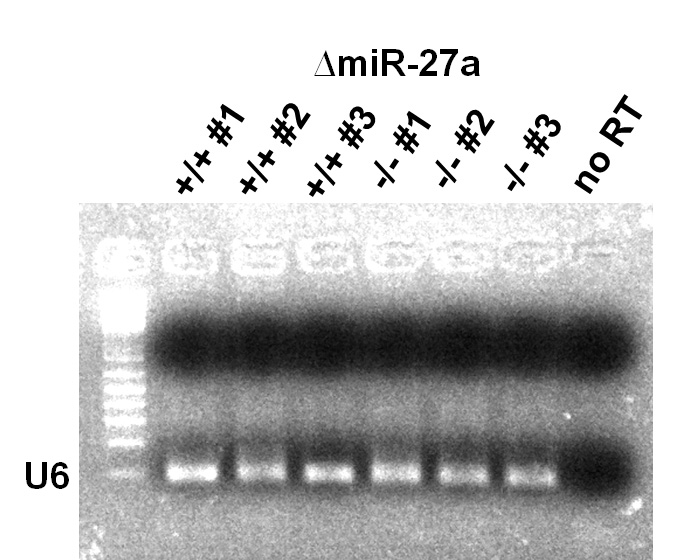

Supplement: Figure 4—source data 8. [file elife-79768-fig4-data8.zip › Figure 4-souce data 8.tif]
