# Supplementary material for: miRNA-27a is essential for bone remodeling by modulating p62-mediated osteoclast signaling
Source: eLife. 2023 Feb 8;12:e79768. doi: 10.7554/eLife.79768 (PMC9946445; doi:10.7554/eLife.79768)
Supplement: Supplementary file 1. [file elife-79768-supp1.docx]

Supplementary Information for

**MicroRNA-27a is essential for bone remodeling by modulating p62-mediated osteoclast signaling**

Shumin Wang^1^, Eri O Maruyama^2^, John Martinez^1^, Justin Lopes^2^, Trunee Hsu^6^, Wencheng Wu^1^, Wei Hsu^1,2,3,4,5,^* and Takamitsu Maruyama^1,2,^*

*Corresponding authors emails

wei.hsu@hsdm.harvard.edu; whsu@forsyth.org; tmaruyama@forsyth.org

**The PDF file includes:**

Primers for RT-PCR analysis of MIR23a~27a~24-2.

Primers for RT-PCR analysis of MIR23a~27a~24-2.

| Name | Primer |
| --- | --- |
| mmu-Mir23a F | GTCTGATCACATTGCCAGGGATTTCC |
| mmu-Mir27a F | GTCTTCACAGTGGCTAAGTTCCGC |
| mmu-Mir24-2 F | GTGGCTCAGTTCAGCAGGAACAG |
| U6 polyA F | CACGCAAATTCGTGAAGCGTTCCAT |
| universal reverse PCR primer | CCAGTCTCAGGGTCCGAGGTATTC |
| miR reverse anchor primer | CGACTCGATCCAGTCTCAGGGTCCGAGGTATTCGATCCTAACCCTCTCCTCGGTATCGAGTCGCACTTTTTTTTTTTTV |
